# Multilocus Sequence Analysis for the Assessment of Phylogenetic Diversity and Biogeography in *Hyphomonas* Bacteria from Diverse Marine Environments

**DOI:** 10.1371/journal.pone.0101394

**Published:** 2014-07-14

**Authors:** Chongping Li, Qiliang Lai, Guizhen Li, Yang Liu, Fengqin Sun, Zongze Shao

**Affiliations:** 1 State Key Laboratory Breeding Base of Marine Genetic Resources, Xiamen, China; 2 Key Laboratory of Marine Genetic Resources, the Third Institute of State Oceanic Administration, Xiamen, China; 3 Collaborative Innovation Center of Marine Biological Resources, Xiamen, China; 4 Key Laboratory of Marine Genetic Resources of Fujian Province, Xiamen, China; University of Minnesota, United States of America

## Abstract

*Hyphomonas*, a genus of budding, prosthecate bacteria, are primarily found in the marine environment. Seven type strains, and 35 strains from our collections of *Hyphomonas*, isolated from the Pacific Ocean, Atlantic Ocean, Arctic Ocean, South China Sea and the Baltic Sea, were investigated in this study using multilocus sequence analysis (MLSA). The phylogenetic structure of these bacteria was evaluated using the 16S rRNA gene, and five housekeeping genes (*leuA*, *clpA*, *pyrH*, *gatA* and *rpoD*) as well as their concatenated sequences. Our results showed that each housekeeping gene and the concatenated gene sequence all yield a higher taxonomic resolution than the 16S rRNA gene. The 42 strains assorted into 12 groups. Each group represents an independent species, which was confirmed by virtual DNA-DNA hybridization (DDH) estimated from draft genome sequences. *Hyphomonas* MLSA interspecies and intraspecies boundaries ranged from 93.3% to 96.3%, similarity calculated using a combined DDH and MLSA approach. Furthermore, six novel species (groups I, II, III, IV, V and XII) of the genus *Hyphomonas* exist, based on sequence similarities of the MLSA and DDH values. Additionally, we propose that the *leuA* gene (93.0% sequence similarity across our dataset) alone could be used as a fast and practical means for identifying species within *Hyphomonas*. Finally, *Hyphomonas*' geographic distribution shows that strains from the same area tend to cluster together as discrete species. This study provides a framework for the discrimination and phylogenetic analysis of the genus *Hyphomonas* for the first time, and will contribute to a more thorough understanding of the biological and ecological roles of this genus.

## Introduction

*Hyphomonas* is a genus of budding, prosthecate bacteria that are primary colonizers of surfaces in the marine environment [1], [2], [3], [4]. The genus *Hyphomonas* was first described by Pongratz [3], [5] in the family *Hyphomonadaceae* of the order *Caulobacterales*. Currently, the genus *Hyphomonas* consists of eight recognized type strains: *Hyphomonas polymorpha* and *Hyphomonas neptunium* [1], *Hyphomonas oceanitis*, *Hyphomonas hirschiana* and *Hyphomonas jannaschiana* [2], *Hyphomonas adhaerens*, *Hyphomonas johnsonii* and *Hyphomonas rosenbergii* [3].

We have isolated many strains of *Hyphomonas* from various oceanic areas over the last eight years (unpublished). Most were isolated from the petroleum-degrading microbial community, indicating that *Hyphomonas* are likely involved in oil degradation. For example, one *Hyphomonas* strain was isolated from a pyrene-enriched consortium of Western Pacific sediment by our laboratory [6], and Zhang *et al.* found others in oil reservoirs [7]. *Hyphomonas* has also been reported in coastal regions such as Heita Bay [8], Milazzo Harbor [9] and the Thames Estuary [10]. However, little is known about the biogeography of the genus *Hyphomonas*, or correlations between their genetic differentiation and geographical distribution.

*Hyphomonas* species delineation based on 16S rRNA gene is difficult because of very high sequence similarities amongst the group [3]. The 16S rRNA gene similarities among type strains of *H. rosenbergii*, *H. hirschiana*, *H. polymorpha* and *H. neptunium* are even at 99.4%. *H. adhaerens* and *H.jannaschiana*, and *H. oceanitis* and *H. johnsonii* also share 99.3% and 98.7% similarity, respectively, between their 16S rRNA gene sequence [3]. According to the commonly used 97.0% sequence similarity cutoff between 16S rRNA gene for species definition [11], [12], the current eight type strains can only be divided into three species.

16S rRNA gene sequence comparison has been the standard for decades for determining bacterial phylogenetic relationships [11], [12]. The advantage of the 16S rRNA gene lies in its universal existence and in its slow rate of evolution. However, it is difficult to differentiate closely related species within some genera such as *Bradyrhizobium* [13], *Streptomyces* [14], *Vibrio* [15], and within the *Bacillus pumilus* group [16]. Various multilocus sequence analysis (MLSA) schemes have been proposed as an alternative to defining bacterial species through time-consuming DNA-DNA hybridization and applied to delineation of diverse taxonomic issues [17], [18], [19], [20], [21], [22], [23].

In this study five housekeeping genes, *leuA* (2-isopropylmalate synthase), *clpA* (ATP-dependent Clp protease), *pyrH* (uridylate kinase), *gatA* (glutamyl-tRNA(Gln) amidotransferase, A subunit) and *rpoD* (RNA polymerase sigma factor), in addition to the 16S rRNA gene, were chosen to analyze the phylogeny of *Hyphomonas* isolates. These housekeeping genes are distributed throughout the chromosome of *H. neptunium* DSM 5154^T^. The phylogenetic diversity based on these genes, and the geographic distribution of *Hyphomonas* bacteria from diverse marine environments was explored, and combined with a MLSA and virtual DNA-DNA hybridization (DDH) analysis evaluated from draft genome sequence.

## Materials and Methods

### Ethics Statement

Detailed information regarding the 42 strains of *Hyphomonas* used in this study is listed in Table 1. Of them, 35 strains were isolated by our laboratory in the past eight years from surface seawater, deep seawater, and deep sediment, with 216L [24] or M2 agar medium [25], sometimes enriching the culture with crude oil prior to isolation. In brief, 25 *Hyphomonas* strains were collected from crude oil enrichment culture according to our previous method [24]. Strain *Hyphomonas* sp. 25B14_1 was isolated from the 1-Chlorohexadecane-degradating bacterial community [26]. Nine strains were obtained through directly plating dilutions of samples without prior enrichment [25]. All isolates have been deposited at the Marine Culture Collection of China (MCCC). Their isolation locations are all in the international sea area (no specific permissions are required), as shown in Figure S1. The eight type strains were purchased from American Type Culture Collection (ATCC) and Deutsche Sammlung von Mikroorganismen und Zellkulturen GmbH (DSMZ).

**Table 1:** Bacterial strains used in this study.

Strains	Original name	MCCC number	Isolation source	Enrichment method	Geographic source	MLSAgroup	Depth (m)
H2	T16B2	1A04387	Sediment	Crude oil	Pacific Ocean	I	−2547
H3	T24B3	1A04464	Sediment	Crude oil	Pacific Ocean	I	−2245
H4	C82AG	1A04485	Seawater	Crude oil	Pacific Ocean	I	−2700
H5	C10AG	1A04665	Seawater	Crude oil	Pacific Ocean	I	−30
H6	C52AD	1A04777	Seawater	Crude oil	Pacific Ocean	I	−500
H7	C57AH	1A04802	Seawater	Crude oil	Pacific Ocean	I	−2695
H8	C68AA	1A04830	Seawater	Crude oil	Pacific Ocean	I	−2192
H9	C6AD	1A04837	Seawater	Crude oil	Pacific Ocean	I	−800
H10	C75AE	1A04859	Seawater	Crude oil	Pacific Ocean	I	−2332
H11	C76AD	1A04862	Seawater	Crude oil	Pacific Ocean	I	−2232
H12	C80AR	1A04889	Seawater	Crude oil	Pacific Ocean	I	−2545
H13	C8AD	1A04910	Seawater	Crude oil	Pacific Ocean	I	−150
H14	C16AH	1A04936	Seawater	Crude oil	Pacific Ocean	I	−1755
H15	L52-1-34	1A05042	Seawater	216L medium	South China Sea	III	0
H16	X1CY54-1-8	1A05051	Seawater	Crude oil	South China Sea	XII	−1
H17	CY54-11-8	1A05059	Seawater	Crude oil	South China Sea	XII	−1000
H18	L53-1-11	1A05080	Seawater	216L medium	South China Sea	III	0
H19	L53-1-40	1A05099	Seawater	216L medium	South China Sea	III	0
H20	C61B20	1A05285	Seawater	Crude oil	Pacific Ocean	I	−1639
H21	C65AK	1A05324	Seawater	Crude oil	Pacific Ocean	I	−1942
H22	C70B2	1A05344	Seawater	Crude oil	Pacific Ocean	I	−1200
H23	C81AK	1A05381	Seawater	Crude oil	Pacific Ocean	I	−2445
H24	C84B2	1A05398	Seawater	Crude oil	Pacific Ocean	I	−1939
H25	C86AW	1A05404	Seawater	Crude oil	Pacific Ocean	I	−2089
H26	GM-8P	1A05653	Seawater	216L medium	South China Sea	XII	−50
H27	1GM01-1C1	1A05819	Seawater	216L medium	South China Sea	III	−812
H28	T5AM	1A06024	Seawater	Crude oil	Pacific Ocean	I	−2484
H29	25B14_1	1A07321	Seawater	1-Chlorohexadecane	Arctic Ocean	II	0
H30	BH-BN04-4	1A07481	Seawater	Crude oil	Arctic Ocean	V	0
H31	22II-20-1h	1A09284	Seawater	216L medium	Atlantic Ocean	IV	−3047
H32	22II1-9F33	1A09376	Seawater	Crude oil	Atlantic Ocean	III	−2238
H36	22II1-22F38	1A09418	Seawater	Crude oil	Atlantic Ocean	IV	−2238
H41	22II-S11e	1A09204	Sediment	216L medium	Atlantic Ocean	IV	−3400
H42	22II-S13e	1A09205	Sediment	216L medium	Atlantic Ocean	IV	−3400
H43	22II-S10j	1A09253	Sediment	216L medium	Atlantic Ocean	IV	−3400
DSM 2665^T^	PS728	1A00471	Nasal mucosa	ND	ND	IX	ND
DSM 5152^T^	VP5	1A00456	Shellfish beds	ND	Pacific Ocean	VIII	−2600
DSM 5154^T^	LE670	1A00409	Seawater	Oligotrophic medium^a^	Mediterranean sea	VIII	ND
DSM 5153^T^	VP2	1A00344	Shellfish beds	ND	Pacific Ocean	XI	−2600
DSM 5155^T^	SCH89	1A00399	ND	Estuarine agar medium^b^	Baltic Sea	VI	ND
ATCC 43964^T^	MHS-2	1A00436	Mud slough	ND	ND	VII	ND
ATCC 43965^T^	MHS-3	1A00391	Mud slough	ND	ND	X	ND

ND, no data; MCCC, Marine Culture Collection of China;

a, refer to Leifson, [61];

b, refer to Weiner et al, [62].

### Cultivation and DNA extraction

All strains were grown on marine agar 2216 medium (BD Difco) at 28°C for 48 h. Genomic DNA was isolated using SBS extraction kit (SBS Genetech Co., Ltd. in Shanghai, China). We note that our re-sequencing of the *H. rosenbergii* ATCC 43869^T^ 16S rRNA gene sequence (under GenBank accession code KF880383) did not match its supposed GenBank accession code (AF082795), and demonstrates that this strain was misidentified in ATCC, and, furthermore, is not deposited in any other culture collection center. Thus, *H. rosenbergii* ATCC 43869^T^ was not included in our study.

### PCR primers and primer design

The universal primers 27F and 1492R were used for amplification of the 16S rRNA gene. The primers for *rpoD* were obtained from a previous study [27]. We designed the *leuA*, *clpA*, *pyrH* and *gatA* primers based on the genome sequences of the thirteen *Hyphomonas* strains. The software package Primer premier 5.0 was used to design and evaluate each pair of primers. Detailed information about the primers used in our study is presented in Table S1.

### PCR amplification and sequencing

PCR amplification of these genes was performed in 50 µL reaction volumes. Each PCR mixture contained 0.5 µL genomic DNA, 2.5 U EasyTaq DNA Polymerase (TransGen Biotech Co., Ltd. in Beijing, China), 4 µL dNTP mixture (2.5 mM of each dNTP), 1 µL each primer (10 µM), 5 µL 10×EasyTaq buffer (Mg^2+^ Plus). The PCR reaction was done in a Biometra T-Professional thermocycler (Biometra; Goettingen, Germany) as follows: an initial denaturation at 95°C for 5 min, 30 cycles of denaturation at 95°C for 30 s, annealing for 30 s at 48°C and extension at 72°C for 50 s, followed by a final extension at 72°C for 10 min. Target PCR products were screened by electrophoresis on a 1% agarose gel and then sequenced using the ABI3730xl platform (Shanghai Majorbio Bio- Pharm Technology Co., Ltd., China). For amplification of *pyrH* and *gatA* genes, primers pyrHf and pyrHr1, gatAf1 and gatAr1 were used to obtain the required fragments from strains H18, H27, H32, H41, H42 and H43. The primers pyrHf and pyrHr, gatAf and gatAr were used to amplify the *pyrH* and *gatA* genes from the remaining strains.

### Sequence analysis

Sequences were examined and assembled using DNAMAN 5.0 software, and then submitted to National Center for Biotechnology Information (NCBI). GenBank accession codes are listed in Table S2. MEGA version 5.05 [28] was used to align and manually trim the sequences and for subsequent phylogenetic analyses, including number of polymorphic sites per gene, and genetic distances using a P-distance model. Phylogenetic trees were constructed in MEGA using the neighbor-joining, maximum parsimony, and maximum likelihood algorithms, all with a 1000 replicate bootstrap resampling. The concatenated sequences of all five protein-coding genes were joined in the following order: *leuA* (774 bp), *clpA* (648 bp), *pyrH* (504 bp), *gatA* (657 bp) and *rpoD* (855 bp).

### Genome sequencing

Twelve representative strains of unique lineages within the genus *Hyphomonas* were selected based on our phylogenetic analysis. Their genomes were sequenced by Shanghai Majorbio Bio-pharm Technology Co., Ltd. (Shanghai, China), using Solexa paired-end (500 bp library) sequencing technology. About 500 Mbp of clean data were generated with an Illumina/Solexa Genome Analyzer IIx (Illumina, SanDiego, CA), reaching approximately 100-fold coverage depth, for each strain. The clean data was assembled using SOAPdenovo2 [29]. GenBank accession codes for these strains genomes are listed in Table S3. The complete genome sequence of *H. neptunium* DSMZ5154^T^ (CP000158.1) was downloaded from NCBI.

### Correlation analysis between similarities of the MLSA and DDH

DNA-DNA hybridization (DDH) estimate values among these 13 genomes were calculated using the genome-to-genome distance calculator website service (GGDC2.0) [30], [31]. Correlation analysis between the similarities of the MLSA and DDH values were performed using the R language, version 3.0.1.

## Results

### 16S rRNA gene analysis

**Figure 1:**
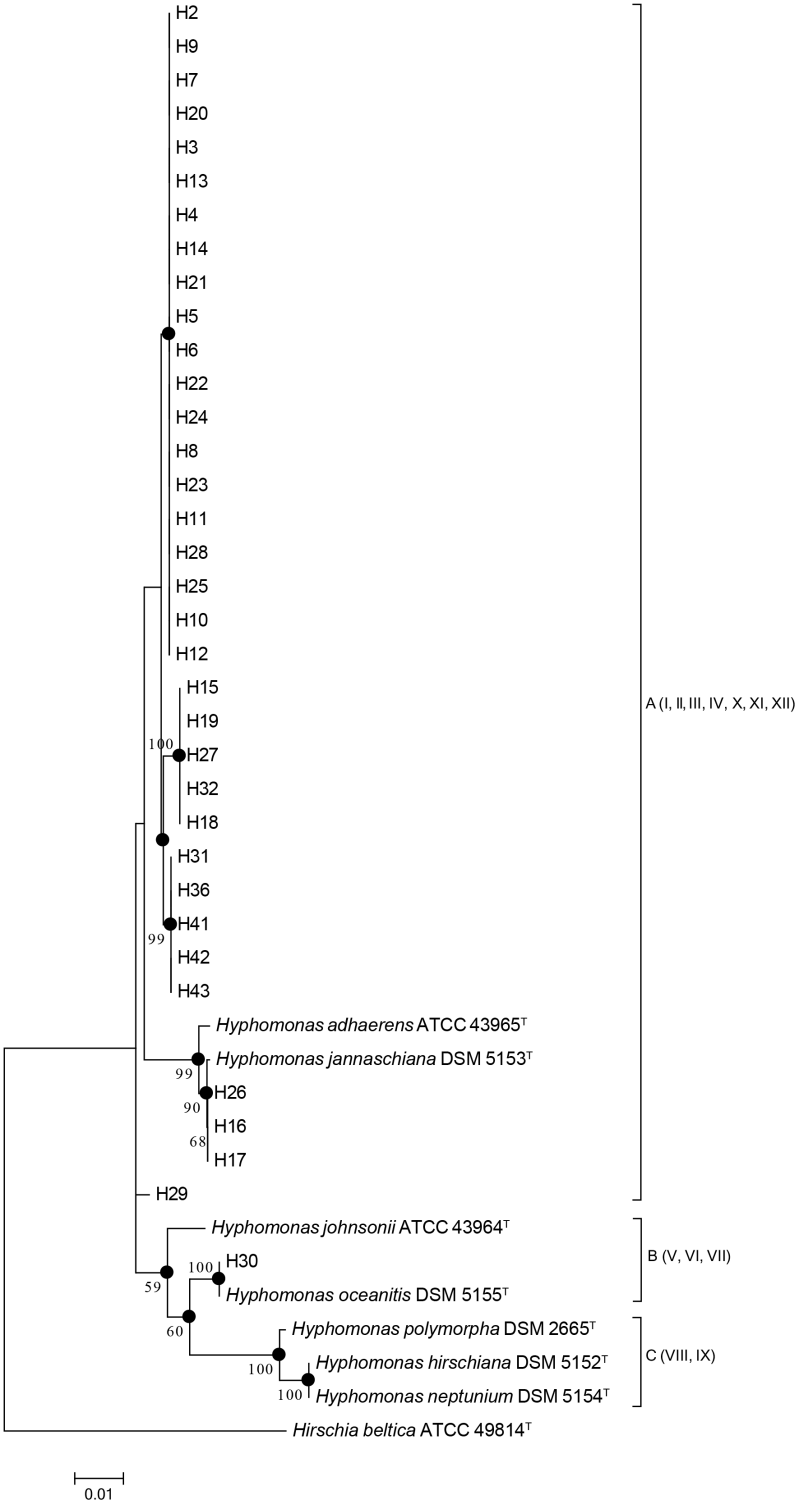
Neighbour-joining tree showing the phylogeny of 42 *Hyphomonas* strains, based on the 16S rRNA gene sequences. Percentage bootstrap values over 50% (1000 replicates) are indicated on internal branches. Filled circles show nodes that were also recovered in maximum-likelihood and maximum-parsimony trees based on the same sequences. Bar, 0.01 nucleotide substitution rate (Knuc) units. *Hirschia beltica* ATCC 49814^T^ (NR_074121) was used as the outgroup.

A sequence similarity cutoff of 97%, according to an often-held species boundary definition [11], [12], segregates our 42 *Hyphomonas* strains into three species, represented by Group A, B and C in the 16S rRNA gene phylogenetic tree presented in Figure 1. Group A was the largest and contained 36 strains, but showed low bootstrap values among the members. The other two groups, B and C, contained three strains each.

Further analysis indicated that genetic distance of the 16S rRNA gene ranged from 0 to 0.042 (Table 2). Intraspecies and interspecies sequence similarities were 100.0% to 100.0%, and 95.8% to 100%, respectively (Table S4). The range of sequence similarities within interspecies comparisons and the crossover of sequence similarities within interspecies and intraspecies comparisons indicate that the 16S rRNA gene is not a suitable phylogenetic marker for *Hyphomonas*. The 16S rRNA gene had 11 alleles. The sequences contained 81 polymorphic sites total, which only comprises 5.7% of all sites in the alignment (Table 2), further demonstrating the high conservation among 16S rRNA genes in *Hyphomonas*.

**Table 2:** Characteristics of the 16S rRNA gene, housekeeping genes and concatenated genes from 42 strains.

Locus	Length (bp)	No. of alleles	Average G+C content (mol%)	Polymorphic sites		P-distance	
				No.	%	Range	Mean
16S rRNA	1419	11	53.6	81	5.7	0–0.042	0.012
*leuA*	774	20	60.6	316	40.8	0–0.224	0.133
*clpA*	648	27	61.5	239	36.9	0–0.198	0.109
*pyrH*	504	27	58.9	211	41.9	0–0.270	0.157
*gatA*	657	17	62.3	270	41.1	0–0.239	0.140
*rpoD*	855	24	59.6	322	37.7	0–0.245	0.122
MLSA	3438	41	60.6	1358	39.5	0–0.217	0.131

### Multilocus sequence analysis

**Figure 2:**
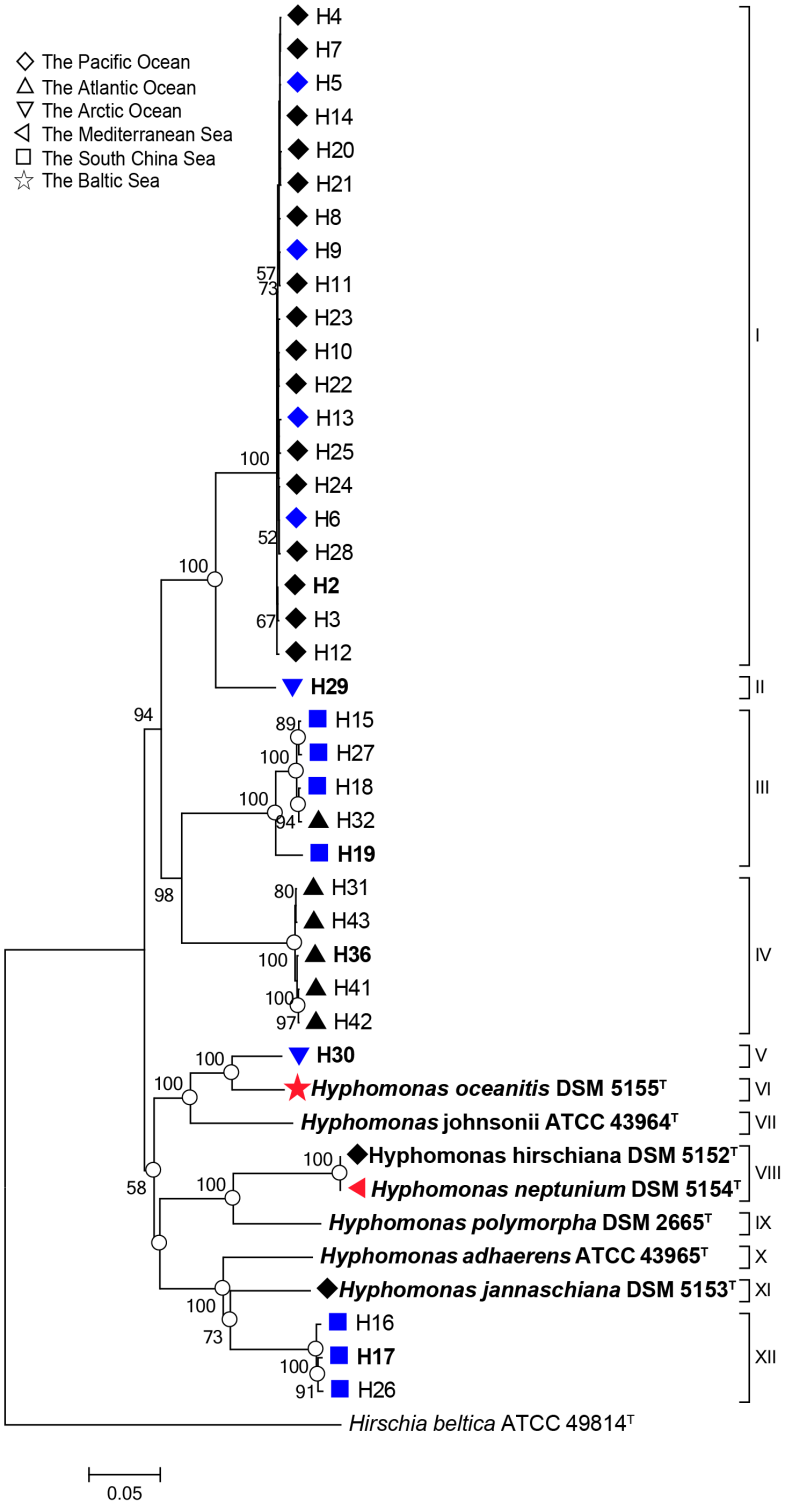
Phylogenetic tree based on concatenated housekeeping genes. Percentage bootstrap values over 50% (1000 replicates) are indicated on internal branches. Blank circles show nodes that were also recovered in maximum-likelihood and maximum-parsimony trees based on the same sequences. Bar, 0.05 nucleotide substitution rate (Knuc) units. *Hirschia beltic*a ATCC 49814^T^ (NC_012982) was used as the outgroup. Water depth is represented by color (0–1000 m, blue color; >1000 m, black color; unknown depth, red color.). No symbol: no detailed information about the source. Bold font strain names indicate their genomes are available.

Another phylogenetic tree was constructed based on the concatenated gene sequences of *leuA*-*clpA*-*pyrH*-*gatA*-*rpoD* (3438 bp) (Figure 2). The topology of this tree demonstrated that these 42 strains could be divided into 12 groups (I–XII). Among these groups, Group I contained 20 strains, which was the largest one. Both Group III and IV contained five apiece, while Group XII contained 3 strains. Interestingly, the two type strains, *H. neptunium* DSM 5154^T^ and *H. hirschiana* DSM 5152^T^, formed Group VIII, implying that they may actually belong to the same species. The remaining groups each consisted of only one strain each. All of these group delineations had relatively high bootstrap values (Figure 2).

Analysis of the correlation between the estimated DDH data and sequence similarities demonstrated that each group likely represents a separate species. The concatenated sequences contained 1358 polymorphic sites, which comprised of 39.5% of all sites in the alignment. The MLSA genetic distance ranged from 0 to 0.217 (Table 2). Furthermore, intraspecies and interspecies sequence similarity comparisons ranged from 96.3% to 100.0% and from 78.3% to 93.3%, respectively, showing an apparent gap between between the intraspecific and interspecific levels (Table S4).

### DDH values and their relationship to the 16S rRNA and housekeeping gene sequence similarity

The draft genome sequences of 12 strains representing each group revealed in our phylogenetic analysis, based on the housekeeping genes and MLSA, were determined. With these genomic data and the complete genome sequence of *H. neptunium* DSM 5154^T^ from GenBank [32], we determined virtual DDH values by pair-wise comparisons among the 13 strains using the website service of GGDC2.0. Estimated DDH values among each group were below the accepted species boundary of 70% [33] (Table S5). Thus, the calculated DDH values confirmed that each group represents an independent species. Furthermore, the high DDH value (100%) between *H. neptunium* DSM 5154^T^ and *H. hirschiana* DSM 5152^T^ also suggests that they belong to the same species, in spite of having different type strain designations.

By plotting the sequence similarities for the 16S rRNA gene, each housekeeping gene and concatenated genes sequence similarities against the estimated DDH values, the sequence similarities threshold relating to species boundary (corresponding to a value of less than 70% DDH relatedness) were obtained (Figure S2). Correlating 16S rRNA gene sequence similarities against DNA−DNA relatedness reconfirmed that the 16S rRNA gene was not an appropriate marker for *Hyphomonas*, as the 70% DDH relatedness corresponds to 100% sequence similarities of the 16S rRNA gene. The sequence similarity delimiting the species boundaries for the housekeeping genes (*leuA*, *clpA*, *pyrH*, *gatA* and *rpoD*) and for the concatenated gene sequences were 93.0%, 96.0%, 93.5%, 91.5%, 95.6% and 93.3%, respectively, which all demonstrated higher taxonomic resolution than the 16S rRNA gene sequence. Moreover, *gatA* possesses the highest resolving power of the five housekeeping genes, followed by *leuA* and then *pryH*. Thus, *Hyphomonas* species discrimination based on MLSA is more reliable and effective than that based on 16S rRNA gene sequence. Based on the sequence similarities of MLSA and DDH values, Group I, II, III, IV, V and XII were allocated to six different novel species.

### Phylogenetic diversity revealed by individual housekeeping genes

Phylogenetic trees based on individual housekeeping genes were also constructed (Figure S3–S7). Although the topologies of these trees are not all identical, the strains within each group in the different trees are the same, and the same as the groups delimited by the concatenated gene sequence. These results imply that these housekeeping genes are adequate for clearly circumscribing species within the genus *Hyphomonas*.

**Figure 3:**
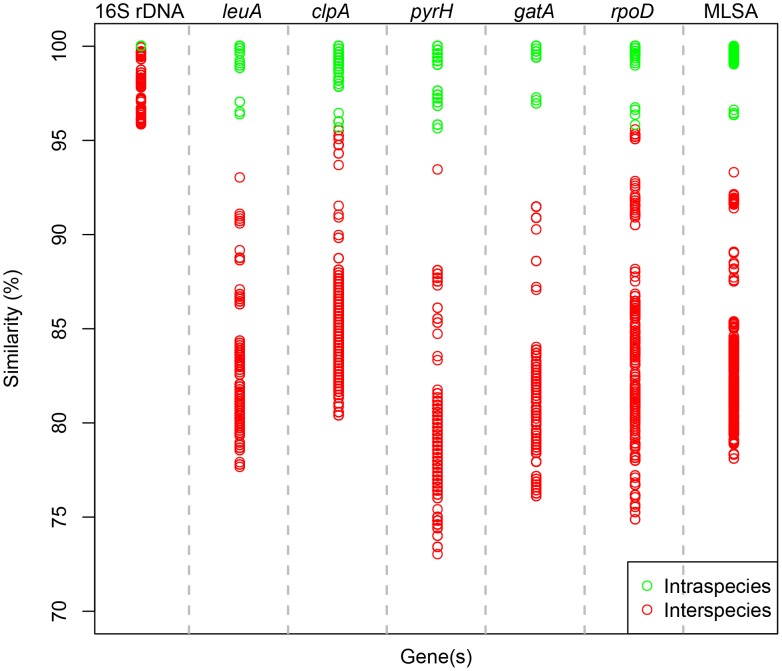
Intraspecies and interspecies similarity ranges of 16S rDNA and housekeeping genes in *Hyphomonas*.

The results of the genetic distance, polymorphic sites were summarized in Table2. Among the five housekeeping genes, *pyrH* had the broadest range of genetic distance range (0–0.270) and the highest percentage of polymorphic sites (41.9%). *leuA* also had a relatively wide genetic distance range (0–0.224) and high percentage of polymorphic sites (40.8%). However, *gatA* exhibited the best taxonomic resolution with genetic distance from 0 to 0.239, and 41.1% polymorphic sites. The remaining housekeeping genes also had a relatively higher percentage of polymorphic sites (>36.9%) than the 16S rRNA gene (5.7%). An apparent gap also existed between the interspecies and intraspecies boundaries in *leuA*, *pryH* and *gatA* (Figure 3). The size of this gap reconfirmed that *gatA* exhibited the highest resolution, and followed by *leuA* and then *pyrH*. We should mention that *leuA* is easier to obtain than *gatA* and *pryH* through PCR amplification.

### Correlation between phylogenetic and geographic distribution

The strains in this study were isolated from various locations across global marine environments, including the Pacific Ocean, Atlantic Ocean, Arctic Ocean, South China Sea, Baltic Sea and the Mediterranean Sea. Twenty strains within Group I were isolated from the Pacific Ocean (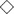 ) (Figure 2). Two other strains, strain DSM 5152^T^ and strain DSM 5153^T^, from the Pacific Ocean formed two independent groups, with strain DSM 5154^T^ segregating along with strain DSM 5153^T^. Four strains from the Atlantic Ocean (△) formed Group IV. All strains clustered in Group III and XII, except for strain H32, were retrieved from the South China Sea (□). Strains H29 and H30 are the only members of Group II and Group V, respectively, and both were from Arctic Ocean (▽). The others from various sites, including the Baltic Sea, and unknown sources, correspond to different groups (VI, VII, IX, X). Strains from the same area tended to cluster together, and strains from different areas tended to form independent groups, indicating that members of this genus inhabiting different geographical areas and evolved independently.

Furthermore, Figure 2 delineates the strains in our phylogenetic tree by different colors according to water depth (0–1000 m, blue; >1000 m, black; unknown depth, red.). However, the distribution of strains in each group presented no obvious pattern regarding water depth. For example, the strains from the upper layer and the deeper layer, in Group I and Group III, clustered together in our analysis. Except for Group XII, as for the remaining groups, the number of strains was not enough to give a persuasive conclusion.

## Discussion

A traditional, wet-lab DDH similarity of ≥70% has been a ‘Gold standard’ for circumscribing species delineation in bacteria for the last several decades [11], [34], [35]. Recent reports have demonstrated that the virtual DDH values calculated by the GGDC web server can adequately mimic wet-lab DDH analysis [30], [36], [37]. Indeed, other computational genome-based methods for replacing wet-lab DDH exist, such as average nucleotide identity (ANI) implementations [38], [39], and the currently accepted ANI threshold for species definition is 95% or higher [39]. However, virtual DDH values are presented on the same scale as wet-lab DDH values. Moreover, virtual DDH analysis has a higher correlation with conventionally determined wet-lab DDH, than do ANI implementations [30], [36], [37]. Furthermore, virtual DDH has been widely applied over many bacterial groups [40], [41], [42], [43]. Previous studies on *Bacillus subtilis* group [44], *Vibrio* [45], *Streptomyces* [46], *Kribbella* [20], indicate that housekeeping genes are a suitable supplement, or an adequate replacement to DNA–DNA hybridization. MLSA has also been successfully applied to several other bacteria, including *Borrelia* [47], *Chlamydiales* [48], *Corynebacterium* [49], *Vibrio* [15] and *Treponema* [50].

In this study, the virtual DDH values among 13 representative strains of the genus *Hyphomonas* were determined. Correlation analysis between the estimated DDH values and individual housekeeping gene (*leuA*, *clpA*, *pyrH*, *gatA* and *rpoD*), concatenated genes sequence similarities demonstrated that the sequence similarities for delimiting species with this *Hyphomonas* dataset range from 91.5% to 96.0%.

The 16S rRNA gene is not an appropriate phylogenetic marker for *Hyphomonas*, as it is far too conserved across the genus. This characteristic has also been observed in other bacteria. The *Bacillus pumilus* group was found to have a 16S rRNA gene sequence similarities among 79 strains ranging from 99.5% to 100% [16]. Other closely related species such as *Bacillus subtilis* group and *Treponema*, were found indistinguishable based on 16S rRNA gene sequence analysis [44], [50]. In this study, some *Hyphomonas* strains with 100% sequence similarities between their 16S rRNA genes shared less than 70% DDH relatedness, reinforcing the conclusion that the 16S rRNA gene has limited power as a phylogenetic marker in some bacterial groups.

Previous reports have indicated that *Pseudomonas* [51], hot spring cyanobacteria [52], *Sulfolobus* [53], and *Myxococcus xanthus* [54] exhibit endemicity at the genotype level. As shown in our MLSA based phylogenetic tree (Fig. 2), *Hyphomonas* strains from the same area tend to cluster together, and strains from different areas tend to form independent groups. Many bacteria tend to distribute similarly, through geographical patterns that parallel lineage assortment [51], [52], [54]. Moreover, studies showed that the local adaptation has been associated with specific environmental conditions including varying sediment composition, light intensity, temperature, and salinity and sulfate concentrations [55], [56], [57], [58]. However, the driving factors that result in the restriction of certain *Hyphomonas* genotypes to particular regions remain unknown.

The genus *Hyphomonas* is a dimorphic, prosthecate bacteria, primarily restricted to, and ubiquitous in the marine environment [4], [59]. Previous reports have shown that *Hyphomonas* are a predominant member of the oil-degradation microbial communities [8], [10]. Genomic analysis of *H. neptunium* DSM 5154^T^ shows that it possesses genes related to the degradation of aromatic compounds [32]. A recent study also reports that an isolate belonging to the genus *Hyphomonas* can degrade carbazole [60]. However, we found that all *Hyphomonas* isolates in our study cannot grow in the presence of oil (unpublished data). Furthermore, we did not find any alkane hydroxylase genes, those responsible for alkane degradation, in the *Hyphomonas* genome sequences that we analyzed. However, three genes are annotated as hydroxylating dioxygenase for polycyclic aromatic hydrocarbons, including two potential naphthalene-degrading hydroxylating dioxygenase (HOC_18389 and HOC_18394,) and one pyrene-degrading related hydroxylating dioxygenase (HOC_16925), in strain *H. oceanitis* DSM 5155^T^. The roles of *Hyphomonas* in oil-degrading communities remain complex and are worthy of further investigation.

In conclusion, a systematic study of *Hyphomonas* diversity was carried out in this study. Using MLSA, based on the *leuA*-*clpA*-*pyrH*-*gatA*-*rpoD* concatenated gene dataset, 42 strains were divided into 12 distinct groups. Furthermore, a MLSA sequence similarity of 93.3% was deemed an appropriate cutoff value for the interspecies *Hyphomonas* boundary using these genes. Among these genes, *gatA* showed the highest taxonomic resolution, followed by *leuA* and *pyrH*. The *leuA* gene, which is the easiest among the three genes to amplify, can be used to identify species within the genus *Hyphomonas* using a 93.0% sequence similarity cutoff, which corresponding to a virtual DDH value of less than 70%. This study should help increase the understanding of the phylogeny, evolutionary history and ecological roles of bacteria in the *Hyphomonas* genus. Polyphasic characterization and comparative genomic analysis among the 12 representative strains used for full genome sequencing await further study.

## Supporting Information

Figure S1
**The map of geographical distribution the 35 strains from various marine environments.** Each red dot represents a strain, some dots overlapped; 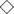, Pacific Ocean; △, Atlantic Ocean;▽, Arctic Ocean; □, South China Sea.(DOCX)

Figure S2 **Comparison of 16S rRNA, individual housekeeping gene (** ***leuA*** **,** ***clpA*** **,** ***pyrH*** **,** ***gatA*** **and** ***rpoD*** **) and concatenated genes sequence similarities and estimated DDH values.** Interspecies comparisons are indicated by red filled circles, whereas intraspecies comparisons are indicated by green filled circles.
(DOCX)

Figure S3 **Phylogenetic tree based on** ***leuA*** **gene.** Percentage bootstrap values over 50% (1000 replicates) are indicated on internal branches. Filled circles show nodes that were also recovered in maximum-likelihood and maximum-parsimony trees based on the same sequences. Bar, 0.05 nucleotide substitution rate (Knuc) units. *Hirschia beltic*a ATCC 49814^T^ (NC_012982) was used as the outgroup.
(DOCX)

Figure S4 **Phylogenetic tree based on** ***clpA*** **gene.** Percentage bootstrap values over 50% (1000 replicates) are indicated on internal branches. Filled circles show nodes that were also recovered in maximum-likelihood and maximum-parsimony trees based on the same sequences. Bar, 0.05 nucleotide substitution rate (Knuc) units. *Hirschia beltic*a ATCC 49814^T^ (NC_012982) was used as the outgroup.
(DOCX)

Figure S5 **Phylogenetic tree based on** ***pyrH*** **gene.** Percentage bootstrap values over 50% (1000 replicates) are indicated on internal branches. Filled circles show nodes that were also recovered in maximum-likelihood and maximum-parsimony trees based on the same sequences. Bar, 0.05 nucleotide substitution rate (Knuc) units. *Hirschia beltic*a ATCC 49814^T^ (NC_012982) was used as the outgroup.
(DOCX)

Figure S6 **Phylogenetic tree based on** ***gatA*** **gene.** Percentage bootstrap values over 50% (1000 replicates) are indicated on internal branches. Filled circles show nodes that were also recovered in maximum-likelihood and maximum-parsimony trees based on the same sequences. Bar, 0.05 nucleotide substitution rate (Knuc) units. *Hirschia beltic*a ATCC 49814^T^ (NC_012982) was used as the outgroup.
(DOCX)

Figure S7 **Phylogenetic tree based on** ***rpoD*** **gene.** Percentage bootstrap values over 50% (1000 replicates) are indicated on internal branches. Filled circles show nodes that were also recovered in maximum-likelihood and maximum-parsimony trees based on the same sequences. Bar, 0.05 nucleotide substitution rate (Knuc) units. *Hirschia beltic*a ATCC 49814^T^ (NC_012982) was used as the outgroup.
(DOCX)

Table S1 **PCR primers used for amplification of 16S rDNA,** ***leuA*** **,** ***clpA*** **,** ***pyrH*** **,** ***gatA*** **and** ***rpoD*** **genes of the genus** ***Hyphomonas*** **.**
(DOCX)

Table S2 **GenBank accession numbers of 6 genes used in this study.**
(DOCX)

Table S3 **The GenBank accession numbers of draft genomes of 12 representatives of the genus** ***Hyphomonas*** **.**
(DOCX)

Table S4 **The similarity variation ranges of the house keeping genes of the 42 strains at intraspecies and interspecies levels.**
(DOCX)

Table S5 **Estimated DDH values among 13 representative strains of the genus** ***Hyphomonas*** **.**
(DOCX)
