# Supplementary material for: Multilocus Sequence Analysis for the Assessment of Phylogenetic Diversity and Biogeography in Hyphomonas Bacteria from Diverse Marine Environments
Source: PLoS One. 2014 Jul 14;9(7):e101394. doi: 10.1371/journal.pone.0101394 (PMC4096408; doi:10.1371/journal.pone.0101394)
Supplement: Figure S1 — The map of geographical distribution the 35 strains from various marine environments. Each red dot represents a strain, some dots overlapped; , Pacific Ocean; △, Atlantic Ocean;▽, Arctic Ocean; □, South China Sea. (DOCX) [file pone.0101394.s001.docx]

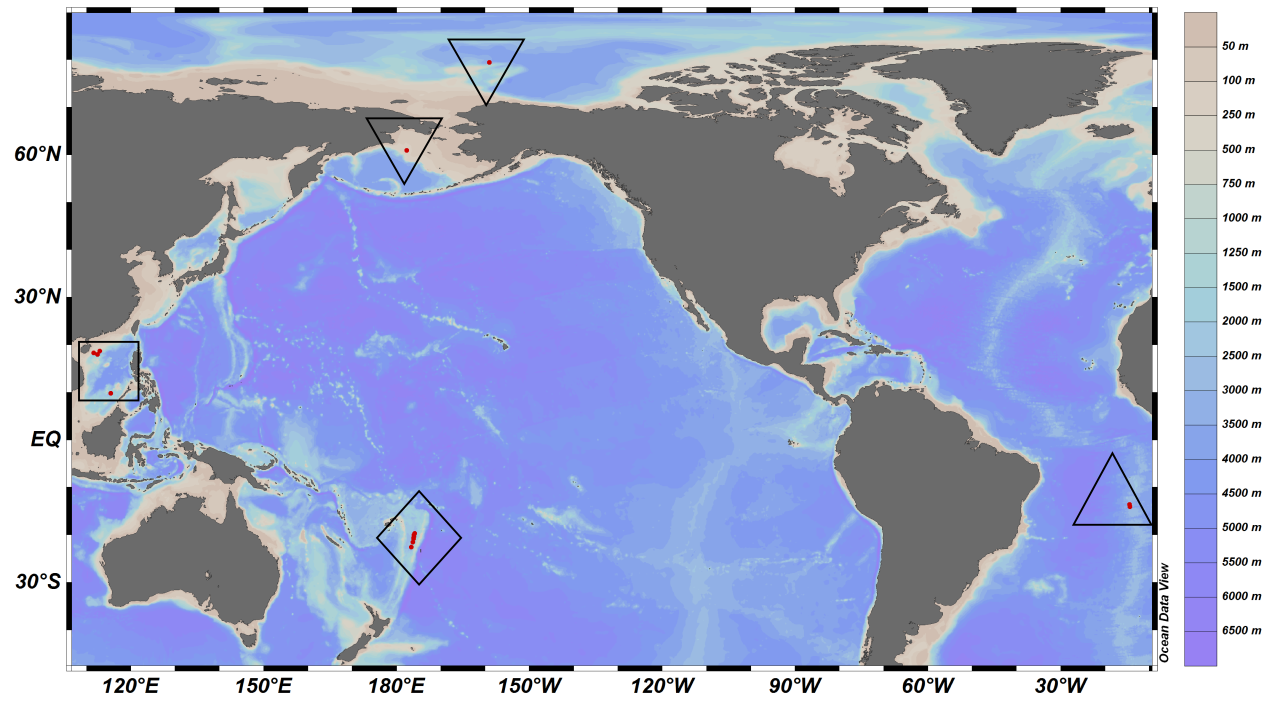


**Figure S1. The map of geographical distribution the 35 strains from various marine environments.** Each red dot represents a strain, some dots overlapped; ◇, Pacific Ocean; △, Atlantic Ocean;▽, Arctic Ocean; □, South China Sea.
