# Supplementary material for: Multilocus Sequence Analysis for the Assessment of Phylogenetic Diversity and Biogeography in Hyphomonas Bacteria from Diverse Marine Environments
Source: PLoS One. 2014 Jul 14;9(7):e101394. doi: 10.1371/journal.pone.0101394 (PMC4096408; doi:10.1371/journal.pone.0101394)
Supplement: Figure S2 — Comparison of 16S rRNA, individual housekeeping gene ( leuA , clpA , pyrH , gatA and rpoD ) and concatenated genes sequence similarities and estimated DDH values. Interspecies comparisons are indicated by red filled circles, whereas intraspecies comparisons are indicated by green filled circles. (DOCX) [file pone.0101394.s002.docx]

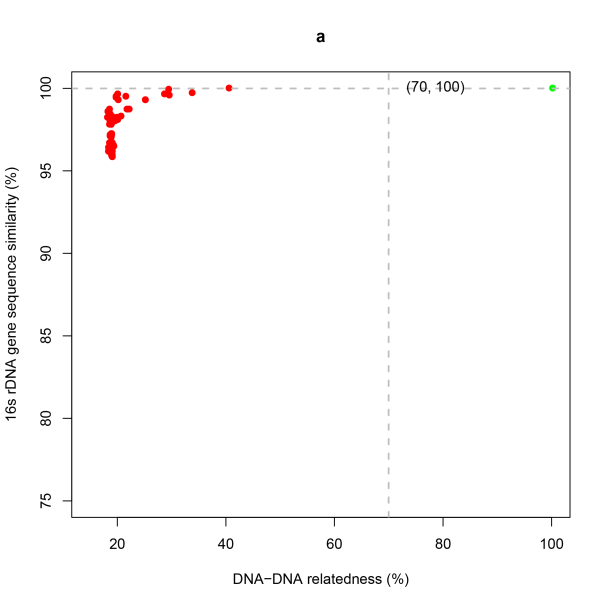

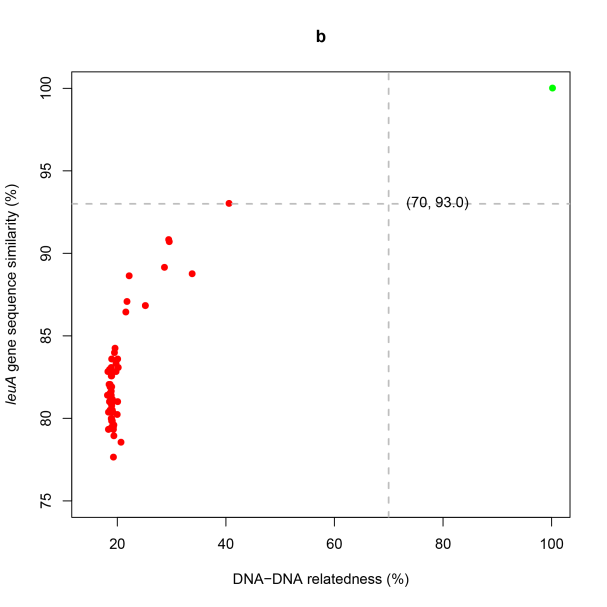


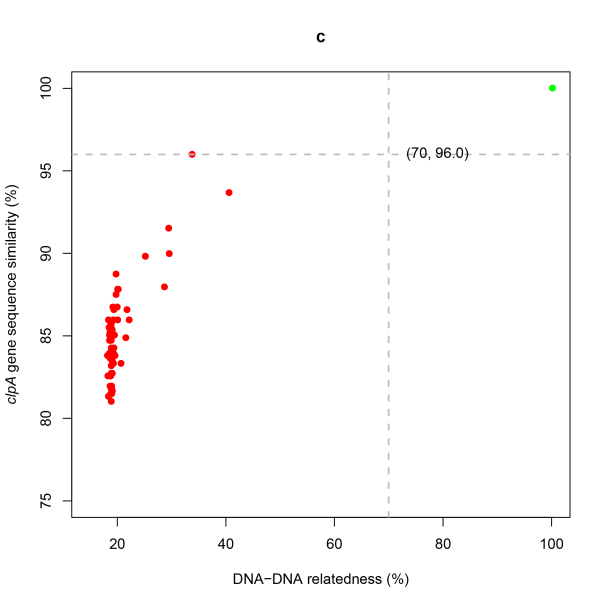

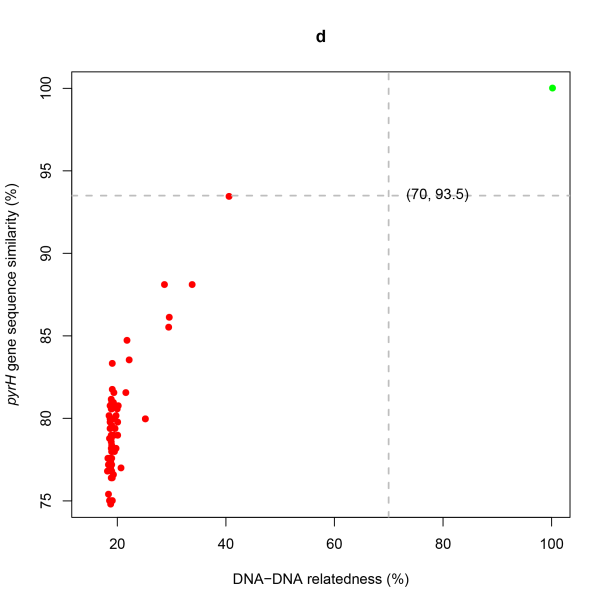


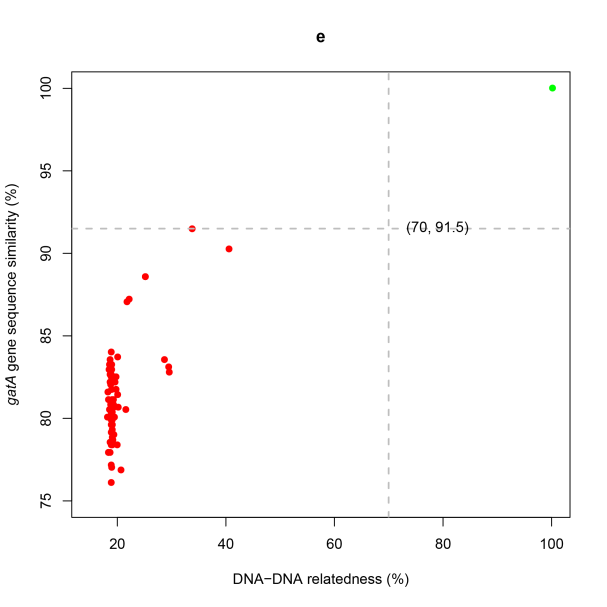

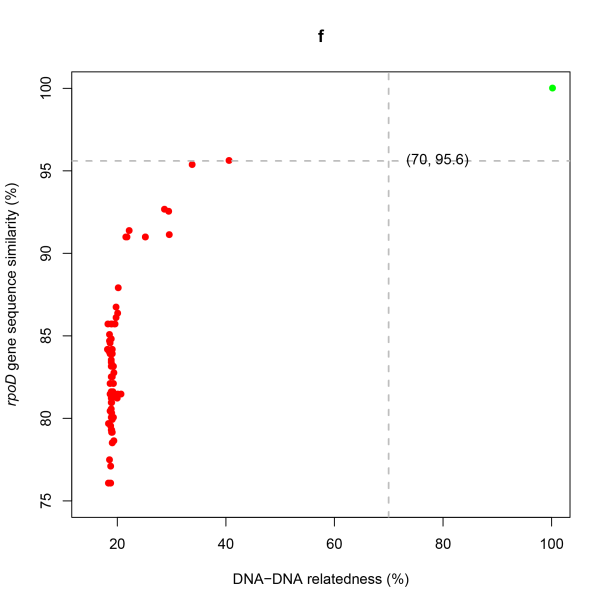


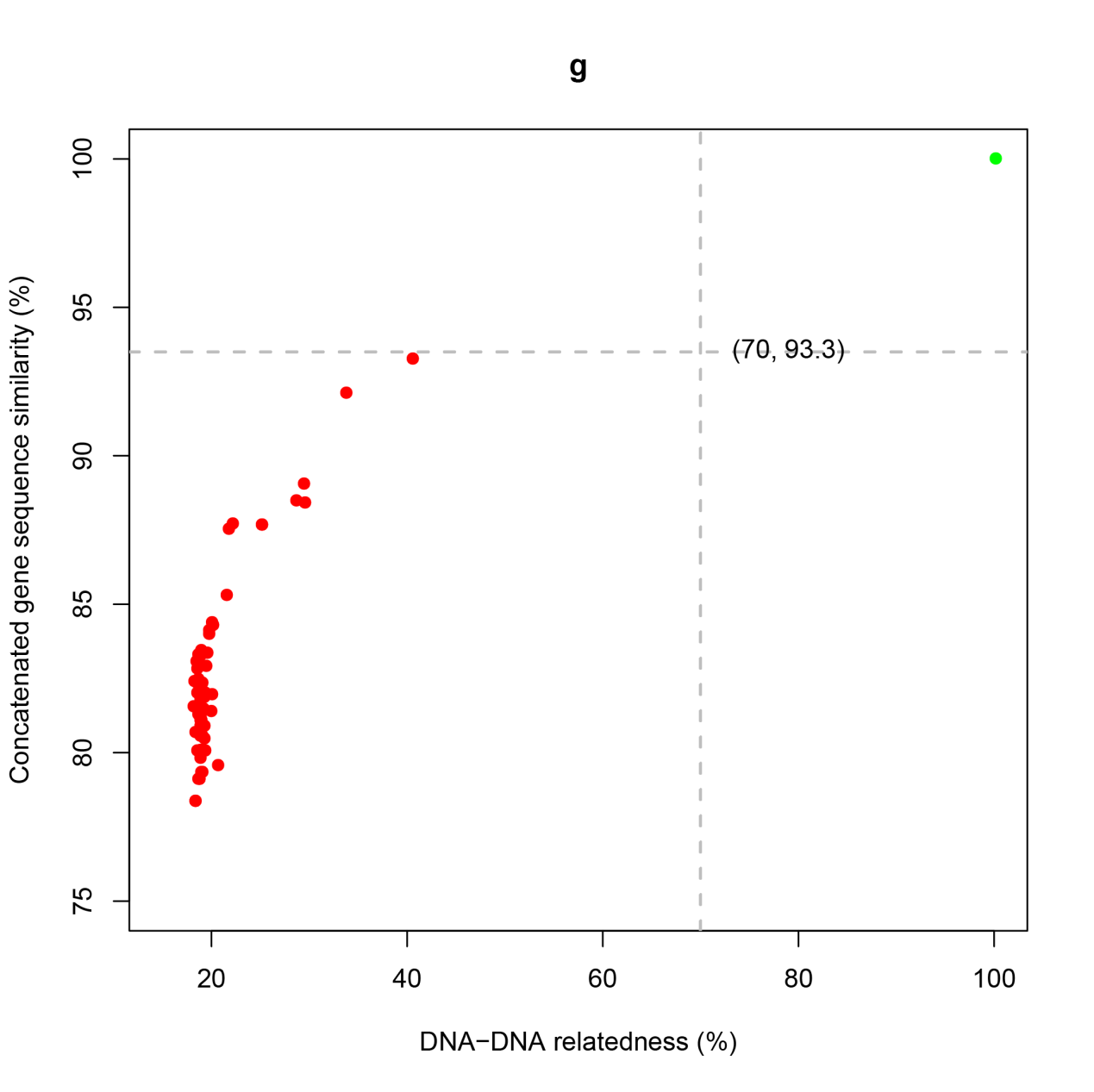


**Figure S2. Comparison of 16S rRNA, individual housekeeping gene (*leuA*, *clpA*, *pyrH*, *gatA* and *rpoD*) and concatenated genes sequence similarities and estimated DDH values respectively.** Interspecies comparisons are indicated by red filled circles, whereas intrrapecies comparisons are indicated by green filled circles. a, 16S rRNA; b, *leuA*; c, *clpA*; d, *pyrH*; e, *gatA*; f, *rpoD*; g, concatenated genes.
