# Supplementary material for: Multilocus Sequence Analysis for the Assessment of Phylogenetic Diversity and Biogeography in Hyphomonas Bacteria from Diverse Marine Environments
Source: PLoS One. 2014 Jul 14;9(7):e101394. doi: 10.1371/journal.pone.0101394 (PMC4096408; doi:10.1371/journal.pone.0101394)
Supplement: Figure S4 — Phylogenetic tree based on clpA gene. Percentage bootstrap values over 50% (1000 replicates) are indicated on internal branches. Filled circles show nodes that were also recovered in maximum-likelihood and maximum-parsimony trees based on the same sequences. Bar, 0.05 nucleotide substitution rate (Knuc) units. Hirschia beltica ATCC 49814T (NC_012982) was used as the outgroup. (DOCX) [file pone.0101394.s004.docx]

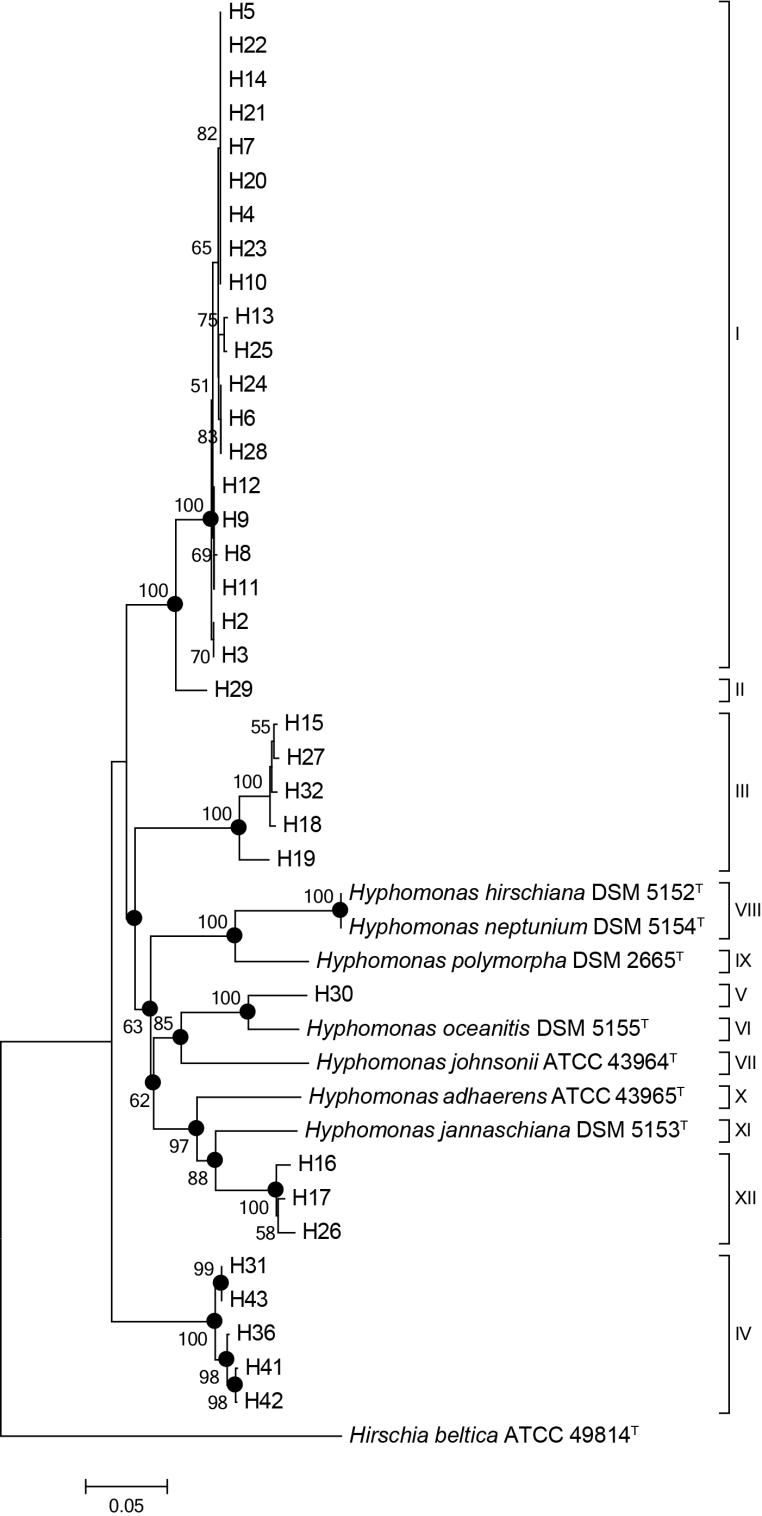


**Figure S4. Phylogenetic tree based on *clpA* gene.** Percentage bootstrap values over 50% (1000 replicates) are indicated on internal branches. Filled circles show nodes that were also recovered in maximum-likelihood and maximum-parsimony trees based on the same sequences. Bar, 0.05 nucleotide substitution rate (Knuc) units. *Hirschia beltic*a ATCC 49814^T^ (NC_012982) was used as the outgroup.
