# Supplementary material for: Multilocus Sequence Analysis for the Assessment of Phylogenetic Diversity and Biogeography in Hyphomonas Bacteria from Diverse Marine Environments
Source: PLoS One. 2014 Jul 14;9(7):e101394. doi: 10.1371/journal.pone.0101394 (PMC4096408; doi:10.1371/journal.pone.0101394)
Supplement: Table S1 — PCR primers used for amplification of 16S rDNA, leuA , clpA , pyrH , gatA and rpoD genes of the genus Hyphomonas . (DOCX) [file pone.0101394.s008.docx]

Table S1. PCR primers used for amplification of 16S rDNA, *leuA*, *clpA*, *pyrH*, *gatA* and *rpoD* genes of the genus *Hyphomonas*

| Gene | Primer name | Sequence (5' to 3') | Annealing temperature (°C) |
| --- | --- | --- | --- |
| 16S rDNA | 27F | AGAGTTTGATCCTGGCTCAG | 48 |
|  | 1492R | ACGGCTACCTTGTTACGACT | 48 |
| *leuA* | leuAf | CAATCGCCCGGCGCCTCCATGA | 48 |
|  | leuAr | TGGATGCCGCTTTCATGCGC | 48 |
| *clpA* | clpAf | CGCCGGTTCCGCAAGAT | 48 |
|  | clpAr | ATGTTGAACAGGTCCGG | 48 |
| *pyrH* | pyrHf | AGTTCGGAATCGATATCCC | 48 |
|  | pyrHr | GARAANACBACRATHGGRAYRTTRTTGTC | 48 |
|  | pyrHr1 | GACTACGATTGGAATGTTGTTG | 48 |
| *gatA* | gatAf | AACATGGACGAGTTCGCCATGG | 48 |
|  | gatAr | AGCACATAGGTCCCGATCA | 48 |
|  | gatAf1 | ATGCTGGGCAAGCTCAAC | 48 |
|  | gatAr1 | CATAAGTGCCAATCATCAGG | 48 |
| *rpoD* | rpoDf | ACGACTGACCCGGTACGCATGTAYATGMGNGARATGGGNACNGT | 48 |
|  | rpoDr | ATAGAAATAACCAGACGTAAGTTNGCYTCNACCATYTCYTTYTT | 48 |
