# Supplementary material for: Multilocus Sequence Analysis for the Assessment of Phylogenetic Diversity and Biogeography in Hyphomonas Bacteria from Diverse Marine Environments
Source: PLoS One. 2014 Jul 14;9(7):e101394. doi: 10.1371/journal.pone.0101394 (PMC4096408; doi:10.1371/journal.pone.0101394)
Supplement: Table S2 — GenBank accession numbers of 6 genes used in this study. (DOCX) [file pone.0101394.s009.docx]

Table S2. GenBank accession numbers of 6 genes used in this study

| Strains | 16S rRNA | *leuA* | *clpA* | *pyrH* | *gatA* | *rpoD* |
| --- | --- | --- | --- | --- | --- | --- |
| H2 | KF863109 | KF863025 | KF863067 | KF863193 | KF862983 | KF863151 |
| H3 | KF863110 | KF863026 | KF863068 | KF863194 | KF862984 | KF863152 |
| H4 | KF863111 | KF863027 | KF863069 | KF863195 | KF862985 | KF863153 |
| H5 | KF863112 | KF863028 | KF863070 | KF863196 | KF862986 | KF863154 |
| H6 | KF863113 | KF863029 | KF863071 | KF863197 | KF862987 | KF863155 |
| H7 | KF863114 | KF863030 | KF863072 | KF863198 | KF862988 | KF863156 |
| H8 | KF863115 | KF863031 | KF863073 | KF863199 | KF862989 | KF863157 |
| H9 | KF863116 | KF863032 | KF863074 | KF863200 | KF862990 | KF863158 |
| H10 | KF863117 | KF863033 | KF863075 | KF863201 | KF862991 | KF863159 |
| H11 | KF863118 | KF863034 | KF863076 | KF863202 | KF862992 | KF863160 |
| H12 | KF863119 | KF863035 | KF863077 | KF863203 | KF862993 | KF863161 |
| H13 | KF863120 | KF863036 | KF863078 | KF863204 | KF862994 | KF863162 |
| H14 | KF863121 | KF863037 | KF863079 | KF863205 | KF862995 | KF863163 |
| H15 | KF863122 | KF863038 | KF863080 | KF863206 | KF862996 | KF863164 |
| H16 | KF863123 | KF863039 | KF863081 | KF863207 | KF862997 | KF863165 |
| H17 | KF863124 | KF863040 | KF863082 | KF863208 | KF862998 | KF863166 |
| H18 | KF863125 | KF863041 | KF863083 | KF863209 | KF862999 | KF863167 |
| H19 | KF863126 | KF863042 | KF863084 | KF863210 | KF863000 | KF863168 |
| H20 | KF863127 | KF863043 | KF863085 | KF863211 | KF863001 | KF863169 |
| H21 | KF863128 | KF863044 | KF863086 | KF863212 | KF863002 | KF863170 |
| H22 | KF863129 | KF863045 | KF863087 | KF863213 | KF863003 | KF863171 |
| H23 | KF863130 | KF863046 | KF863088 | KF863214 | KF863004 | KF863172 |
| H24 | KF863131 | KF863047 | KF863089 | KF863215 | KF863005 | KF863173 |
| H25 | KF863132 | KF863048 | KF863090 | KF863216 | KF863006 | KF863174 |
| H26 | KF863133 | KF863049 | KF863091 | KF863217 | KF863007 | KF863175 |
| H27 | KF863134 | KF863050 | KF863092 | KF863218 | KF863008 | KF863176 |
| H28 | KF863135 | KF863051 | KF863093 | KF863219 | KF863009 | KF863177 |
| H29 | KF863136 | KF863052 | KF863094 | KF863220 | KF863010 | KF863178 |
| H30 | KF863137 | KF863053 | KF863095 | KF863221 | KF863011 | KF863179 |
| H31 | KF863138 | KF863054 | KF863096 | KF863222 | KF863012 | KF863180 |
| H32 | KF863139 | KF863055 | KF863097 | KF863223 | KF863013 | KF863181 |
| H36 | KF863140 | KF863056 | KF863098 | KF863224 | KF863014 | KF863182 |
| H41 | KF863141 | KF863057 | KF863099 | KF863225 | KF863015 | KF863183 |
| H42 | KF863142 | KF863058 | KF863100 | KF863226 | KF863016 | KF863184 |
| H43 | KF863143 | KF863059 | KF863101 | KF863227 | KF863017 | KF863185 |
| DSM 2665^T^ | KF863144 | KF863060 | KF863102 | KF863228 | KF863018 | KF863186 |
| DSM 5152^T^ | KF863145 | KF863061 | KF863103 | KF863229 | KF863019 | KF863187 |
| DSM 5154^T^ | KF863147 | KF863063 | KF863105 | KF863231 | KF863021 | KF863189 |
| DSM 5153^T^ | KF863146 | KF863062 | KF863104 | KF863230 | KF863020 | KF863188 |
| DSM 5155^T^ | KF863148 | KF863064 | KF863106 | KF863232 | KF863022 | KF863190 |
| ATCC 43964^T^ | KF863149 | KF863065 | KF863107 | KF863233 | KF863023 | KF863191 |
| ATCC 43965^T^ | KF863150 | KF863066 | KF863108 | KF863234 | KF863024 | KF863192 |
