# Supplementary material for: Multilocus Sequence Analysis for the Assessment of Phylogenetic Diversity and Biogeography in Hyphomonas Bacteria from Diverse Marine Environments
Source: PLoS One. 2014 Jul 14;9(7):e101394. doi: 10.1371/journal.pone.0101394 (PMC4096408; doi:10.1371/journal.pone.0101394)
Supplement: Table S3 — The GenBank accession numbers of draft genomes of 12 representatives of the genus Hyphomonas . (DOCX) [file pone.0101394.s010.docx]

Table S3. The GenBank accession numbers of draft genomes of 12 representatives of the genus *Hyphomonas*

| Strains | MCCC number | Species | Group | GenBank accession number of genome |
| --- | --- | --- | --- | --- |
| H2 | 1A04387 | *Hyphomonas* sp. | I | AWFA00000000 |
| H17 | 1A05059 | *Hyphomonas* sp. | XII | AWFD00000000 |
| H19 | 1A05099 | *Hyphomonas* sp. | III | AWFI00000000 |
| H29 | 1A07321 | *Hyphomonas* sp. | II | AWFF00000000 |
| H30 | 1A07481 | *Hyphomonas* sp. | V | AWFG00000000 |
| H36 | 1A09418 | *Hyphomonas* sp. | IV | AWFH00000000 |
| DSM 2665^T^ | 1A00471 | *Hyphomonas polymorpha* | IX | ARYM00000000 |
| DSM 5152^T^ | 1A00456 | *Hyphomonas hirschiana* | VIII | ARYI00000000 |
| DSM 5153^T^ | 1A00344 | *Hyphomonas jannaschiana* | XI | ARYJ00000000 |
| DSM 5155^T^ | 1A00399 | *Hyphomonas oceanitis* | VI | ARYL00000000 |
| ATCC 43964^T^ | 1A00436 | *Hyphomonas johnsonii* | VII | ARYK00000000 |
| ATCC 43965^T^ | 1A00391 | *Hyphomonas adhaerens* | X | ARYH00000000 |
