# Supplementary material for: Multilocus Sequence Analysis for the Assessment of Phylogenetic Diversity and Biogeography in Hyphomonas Bacteria from Diverse Marine Environments
Source: PLoS One. 2014 Jul 14;9(7):e101394. doi: 10.1371/journal.pone.0101394 (PMC4096408; doi:10.1371/journal.pone.0101394)
Supplement: Table S4 — The similarity variation ranges of the house keeping genes of the 42 strains at intraspecies and interspecies levels. (DOCX) [file pone.0101394.s011.docx]

Table S4. The similarity variation ranges of the house keeping genes of the 42 strains at intraspecies and interspecies levels

|  | Similarities (%) | |
| --- | --- | --- |
|  | Intraspecies | Interspecies |
| 16S rDNA | 100-100 | 95.8-100 |
| *leuA* | 96.4-100 | 77.6-93.0 |
| *clpA* | 95.7-100 | 80.2-96.0 |
| *pyrH* | 95.6-100 | 73.0-93.5 |
| *gatA* | 97.0-100 | 76.1-91.5 |
| *rpoD* | 96.4-100 | 75.5-95.6 |
| MLSA | 96.3-100 | 78.3-93.3 |
