# Supplementary material for: Multilocus Sequence Analysis for the Assessment of Phylogenetic Diversity and Biogeography in Hyphomonas Bacteria from Diverse Marine Environments
Source: PLoS One. 2014 Jul 14;9(7):e101394. doi: 10.1371/journal.pone.0101394 (PMC4096408; doi:10.1371/journal.pone.0101394)
Supplement: Table S5 — Estimated DDH values among 13 representative strains of the genus Hyphomonas . (DOCX) [file pone.0101394.s012.docx]

Table S5. Estimated DDH values among 13 representative strains of the genus *Hyphomonas*

| Strains | | Species | Group | 1 | | 2 | | 3 | | 4 | | 5 | | 6 | | 7 | | 8 | | 9 | | 10 | | 11 | | 12 | |
| --- | --- | --- | --- | --- | --- | --- | --- | --- | --- | --- | --- | --- | --- | --- | --- | --- | --- | --- | --- | --- | --- | --- | --- | --- | --- | --- | --- |
|  |  |  |  | DDH | C.I | DDH | C.I. | DDH | C.I. | DDH | C.I. | DDH | C.I. | DDH | C.I. | DDH | C.I. | DDH | C.I. | DDH | C.I. | DDH | C.I. | DDH | C.I. | DDH | C.I. |
| 1 | H2 | *Hyphomonas* sp. | I |  |  |  |  |  |  |  |  |  |  |  |  |  |  |  |  |  |  |  |  |  |  |  |  |
| 2 | H17 | *Hyphomonas* sp. | XII | 19.8 | 2.8 |  |  |  |  |  |  |  |  |  |  |  |  |  |  |  |  |  |  |  |  |  |  |
| 3 | H19 | *Hyphomonas* sp. | III | 19.6 | 3.0 | 18.7 | 2.7 |  |  |  |  |  |  |  |  |  |  |  |  |  |  |  |  |  |  |  |  |
| 4 | H29 | *Hyphomonas* sp. | II | 33.6 | 3.1 | 19.0 | 2.8 | 20.0 | 3.0 |  |  |  |  |  |  |  |  |  |  |  |  |  |  |  |  |  |  |
| 5 | H30 | *Hyphomonas* sp. | V | 18.5 | 2.8 | 18.7 | 2.8 | 19.4 | 2.8 | 18.8 | 2.8 |  |  |  |  |  |  |  |  |  |  |  |  |  |  |  |  |
| 6 | H36 | *Hyphomonas* sp. | IV | 19.9 | 3.0 | 20.5 | 2.8 | 21.4 | 3.0 | 19.6 | 3.0 | 18.4 | 2.7 |  |  |  |  |  |  |  |  |  |  |  |  |  |  |
| 7 | DSM 2665^T^ | *Hyphomonas polymorpha* | IX | 18.6 | 2.7 | 19.2 | 2.7 | 18.4 | 2.6 | 18.2 | 2.7 | 18.7 | 2.7 | 18.6 | 2.6 |  |  |  |  |  |  |  |  |  |  |  |  |
| 8 | DSM 5152^T^ | *Hyphomonas hirschiana* | VIII | 18.9 | 2.6 | 18.8 | 2.7 | 18.2 | 2.6 | 18.8 | 2.6 | 18.7 | 2.7 | 18.5 | 2.6 | 25.0 | 3.0 |  |  |  |  |  |  |  |  |  |  |
| 9 | DSM 5154^T^ | *Hyphomonas neptunium* | VIII | 18.9 | 2.6 | 18.9 | 2.7 | 18.2 | 2.6 | 18.8 | 2.6 | 18.8 | 2.7 | 18.6 | 2.6 | 25.0 | 3.0 | 100 | Inf |  |  |  |  |  |  |  |  |
| 10 | DSM 5153^T^ | *Hyphomonas jannaschiana* | XI | 19.1 | 2.8 | 29.3 | 3.1 | 18.8 | 2.7 | 19.2 | 2.8 | 18.9 | 2.8 | 19.1 | 2.7 | 19.1 | 2.7 | 18.7 | 2.7 | 18.8 | 2.7 |  |  |  |  |  |  |
| 11 | DSM 5155^T^ | *Hyphomonas oceanitis* | VI | 18.3 | 2.8 | 18.8 | 2.8 | 19.3 | 2.7 | 18.7 | 2.8 | 40.4 | 3.2 | 18.7 | 2.7 | 18.8 | 2.7 | 18.5 | 2.7 | 18.6 | 2.7 | 18.9 | 2.8 |  |  |  |  |
| 12 | ATCC 43964^T^ | *Hyphomonas johnsonii* | VII | 18.5 | 2.7 | 18.7 | 2.8 | 18.1 | 2.7 | 18.4 | 2.7 | 22.0 | 3.0 | 18.0 | 2.7 | 18.7 | 2.7 | 18.7 | 2.7 | 18.8 | 2.7 | 18.7 | 2.8 | 21.6 | 3.0 |  |  |
| 13 | ATCC 43965^T^ | *Hyphomonas adhaerens* | X | 18.9 | 2.8 | 29.4 | 3.1 | 18.5 | 2.7 | 19.9 | 2.8 | 18.9 | 2.8 | 18.7 | 2.7 | 18.9 | 2.7 | 18.9 | 2.7 | 18.9 | 2.7 | 28.5 | 3.1 | 18.7 | 2.7 | 18.5 | 2.8 |

C.I : Model-based confidence intervals
